# Surface Estimation for Microwave Imaging

**DOI:** 10.3390/s17071658

**Published:** 2017-07-19

**Authors:** Douglas Kurrant, Jeremie Bourqui, Elise Fear

**Affiliations:** Schulich School of Engineering, University of Calgary, Calgary, AB T2N 1N4, Canada; bourquij@ucalgary.ca (J.B.); fear@ucalgary.ca (E.F.)

**Keywords:** surface reconstruction, microwave breast imaging, performance metrics, radar

## Abstract

Biomedical imaging and sensing applications in many scenarios demand accurate surface estimation from a sparse set of noisy measurements. These measurements may arise from a variety of sensing modalities, including laser or electromagnetic samples of an object’s surface. We describe a state-of-the-art microwave imaging prototype that has sensors to acquire both microwave and laser measurements. The approach developed to translate sparse samples of the breast surface into an accurate estimate of the region of interest is detailed. To evaluate the efficacy of the method, laser and electromagnetic samples are acquired by sensors from three realistic breast models with varying sizes and shapes. A set of metrics is developed to assist with the investigation and demonstrate that the algorithm is able to accurately estimate the shape of a realistic breast phantom when only a sparse set of data are available. Moreover, the algorithm is robust to the presence of measurement noise, and is effective when applied to measurement scans of patients acquired with the prototype.

## 1. Introduction

Medical microwave imaging involves extracting information about the internal structure of tissues using microwave fields transmitted through or reflected from the tissues [1]. As tissues have microwave frequency properties that vary with water content, maps of tissue distributions showing dielectric properties (e.g., [2]), or dielectric property discontinuities (e.g., [3]) may be created. Proposed applications include breast health monitoring [4,5], stroke classification [6], bone health assessment [7], and soft tissue imaging (forearm) [8]. In order to create microwave (MW) images, sophisticated sensing systems have been created. These systems enable the tissues to be illuminated with electromagnetic (EM) fields, and detect fields reflected from and transmitted through the tissues.

For these systems, the present accepted best practice used to conduct pre-clinical trials of near-field microwave imaging is for the patient to lie in the prone position on an examination table with the breast suspended through a hole in the table. Moreover, the breast is either suspended freely in an immersion medium that fills a measurement chamber (e.g., [9,10]), or is placed in a matching Eccostock ceramic cup (e.g., [11,12,13]). There are no outside forces applied to the breast beyond those resulting from the immersion liquid or cup. The chest wall of the patient is supported by the table (hard surface covered in foam) incorporated into these systems. Supporting the chest wall minimizes respiration related movement of the breast position within the measurement chamber [3]. Hence, once the patient has settled into a position on the prototype, the breast’s position and surface shape within the measurement chamber are assumed fixed over the duration of the measurement scan.

Given that shape and location of the breast are fixed within the measurement chamber, the primary purpose of the technique described in this paper is to acquire an estimate of the surface of the breast. As the breast is immersed in liquid or positioned in a ceramic cup, estimating the location of the surface in the scanner is challenging.

Acquiring the shape of the breast surface within the measurement chamber is necessary to form effective images. The skin surface bounds the breast interior that includes skin, fat and fibroglandular tissues; the immersion medium or ceramic cup is external to this boundary. The goal of the imaging algorithm is to image the breast interior (i.e., the region of interest). Hence, identifying the breast surface allows the imaging domain to be defined. Clinical imaging modalities such as CT and MRI do not need an estimate of the skin surface for image reconstruction. Ultrasound typically scans a localized region and an estimate of the entire breast surface is not required. Conversely, for MW imaging—either radar imaging or microwave tomography—measurements of the surface are needed to define bounds for the image. For example, identifying the surface is required for radar-based microwave imaging systems in order to calculate path lengths between the sensor and surface, as well as in the various tissues. These path lengths and estimates of the velocities over the paths are used to evaluate time delays associated with points in the image domain that are used for image formation ([3,11,12,13], for example). For MW tomography, constraining the imaging domain to within the breast surface substantially improves image quality and also serves to encourage convergence to the actual solution (e.g., [14,15]).

Surface estimates may also be used to enable flexible positioning of sensors. As medical microwave imaging is a relatively new approach, optimal positioning of sensors has not been defined. In the context of microwave breast imaging, the study presented in [16] demonstrated that sensor positions tailored to the geometry of the breast provide greater responses from targets, superior object coverage and improved images compared to cylindrical or hemispherical scan patterns. We refer to this collection of sensor positions as a patient specific, or more generally, an object specific data acquisition surface. Forming this data acquisition surface is facilitated by the flexible positioning capabilities of our monostatic radar prototype, as well as methods that provide accurate estimates of the breast surface.

Finally, in the context of the ceramic cup used in the clinical studies described in [11,12], several ceramic inserts are designed to accommodate patients with different breast sizes. A small amount of matching medium is applied to fill any gaps between the breast and cup. Air or excess fluid can be trapped between the insert and the patient’s breast. Therefore, an estimation of the surface from the electromagnetic measurements may be used to identify the location of any badly fitted breast area and allow the system user to reposition the patient’s breast in the cup/insert accordingly. For this measurement scenario, the use of EM measurements may be used to form an estimate of the breast surface (e.g., [17,18,19]).

At the University of Calgary, we have developed two prototype systems incorporating laser sensors. The first monostatic radar-based prototype developed at the University of Calgary was used for initial pre-clinical trials [9]. It implemented a cylindrical scan pattern through the rotation of the tank and vertical movement of the arm to which the sensors are attached. Using the cylindrical scan pattern, the sensor’s axis and tip were fixed in the same position throughout the measurement scan. Laser data were collected at the set of scan locations where microwave measurements were acquired; both sensors were stationary [20]. The result was a set of static surface samples forming a point cloud. The tessellation algorithm reported in [21] was applied directly to this point cloud to produce a surface mesh. The surface information was only used for image formation.

The second monostatic radar-based prototype uses patient-specific EM sensor positioning [22]. To implement this positioning, the breast surface is estimated from samples acquired with a laser that scans rapidly over the surface. Acquiring data in this manner leads to two significant consequences. First, the data are noisy (e.g., perturbations of the robotic arm as it moves rapidly over the surface). Second, a surface mesh cannot be created by applying a tessellation algorithm directly to the data. The surface estimation technique described in this paper enables patient-specific sensor positioning, and provides a means to form a surface mesh used for image reconstruction.

A brief outline of the surface reconstruction procedure was first introduced in [23]. We extend this original work by furnishing a detailed description of our state-of-the-art prototype, and the surface estimation algorithm that provides accurate results from sparse, noisy data. We demonstrate the robustness of this algorithm by applying it to experimental data generated with anthropomorphic breast phantoms. The reconstruction technique is also extended from [23] to operate on data generated by either laser or electromagnetic sensors. This capability demonstrates its flexibility to be able to reconstruct surfaces from data generated from a variety of modalities.

The prototype, as well as methods developed to estimate the surface are described in Section 2. The approach used to measure the accuracy of the surface fit to laser and electromagnetic measurements is also described in Section 2. The metrics are applied to estimated profiles and surfaces to demonstrate the efficacy of the algorithms in Section 3. Importantly, the utility of the algorithm is demonstrated in a clinical setting in Section 3 by applying it to patient data. Section 4 summarizes and concludes the study.

## 2. Materials and Methods

First, descriptions of the prototype and the approach to data collection are provided. Next, we present the procedure to obtain an accurate estimate of the surface along a profile of the object by fitting a model to the surface samples. The second algorithm presented reconstructs a model of the breast surface by synthesizing the individual profiles formed with the first algorithm. Finally, the models and metrics used to evaluate the algorithms are described.

### 2.1. Prototype and Data Acquisition

Several prototype microwave breast imaging systems incorporate multiple sensing modalities to provide estimates of the breast surface. For the microwave tomography system described in [24], a high-resolution scanning system consists of two laser line generators and a small charge-coupled device (CCD) camera mounted concentrically on a gantry that rotates around a microwave measurement tank. Since the sensors are outside the measurement tank, various calibration methods, including traditional ray tracing and image registration approaches, are required for optimizing the accuracy of the scanner in the presence of the optical distortions. The system acquires a significantly denser set of surface samples than the prototype described in this paper, and the results presented in [24] (e.g., Figure 10 on page 3109) demonstrate very high quality estimated surfaces. However, this is achieved with a sensing system located outside of the measurement tank. To collect the microwave data, an array of antennas attached to a ring is scanned vertically through the tank, so the surface is used for image reconstruction and not sensor positioning.

**Figure 1:**
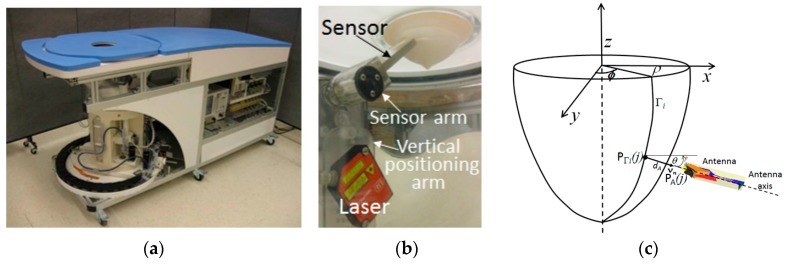
(**a**) Prototype with capability to position sensor with 4 degrees of freedom. (**b**) Laser and microwave sensor connected to vertical positioning arm. (**c**) Robotic arm is incrementally rotated around breast by angle *ϕ* and moves laser in vertical direction to estimate breast profile *Γ_i_*. Sensor arm controls angle of inclination *θ* and distance *d_A_* of aperture *P_A_* from point on breast surface *P_Γi_*.

At the University of Calgary, we have developed a prototype system for monostatic, radar-based breast imaging [22]. The absorption of microwave energy in tissues has been evaluated for this prototype, and found to be below safety guidelines for public exposures [25]. The prototype consists of a measurement chamber filled with canola oil and has been designed with the requirement that the robotic-arm controlling the position of the custom-designed ultra-wideband electromagnetic sensor [26] relative to the breast be located in the measurement chamber. This requirement is imposed on the design so that patient specific positioning of the sensor is achieved. In particular, the sensor tip can be positioned at a specific distance from the patient’s skin and the sensor’s orientation relative to the skin surface takes into account the curvature of the breast surface. To achieve this, the curvature and location of the breast within the measurement chamber is acquired with a laser. The laser is attached along with the electromagnetic sensor to the arm (Figure 1), rather than locating the laser outside the measurement chamber, as is done with the system in [24]. This significantly simplifies the design, as the same robotic arm can be used to move both the electromagnetic sensor and laser.

The arm moves vertically in the tank at a distance from the object in order to measure its profile (*Γ_i_*) with laser samples. A linear transformation is applied to the data generated by the laser (OptoNCDT1300-100, Microepsion, Ortenburg, Germany) to map these data to corresponding cylindrical coordinates [27]. A calibration factor is applied to account for the canola oil that fills the tank. Next, the antenna is positioned at the appropriate data collection location. The prototype allows 4 degrees of freedom in positioning to provide the capability to adapt to different breast sizes and shapes while orienting the sensor approximately normal to the breast skin (Figure 1b). A camera is located outside the tank to observe the breast position within the chamber and to confirm appropriate antenna location and orientation. The approaches to data acquisition result in specific performance demands for the algorithms that are used to estimate profiles and the object surface.

As pointed out in [20], a laser scanning technique offers high accuracy and resolution compared to microwave-based methods. However, achieving these benefits requires that the surface scan be performed concurrently with the microwave scan. Concurrent data collection is achieved with a two-step procedure.

First, surface samples are acquired with a simple laser line projection along a single profile. The laser dynamically acquires surface measurements; surface samples are recorded on-the-go as the laser that is attached to the robotic arm rapidly moves over the surface of the object. The location of the arm is used as a reference in order to accurately transform each surface sample to a point in 3D space within the measurement chamber. Since this arm is moving, its speed and acceleration through space are taken into account when calculating its position.

In the second step, a single profile (i.e., a curve) is estimated from the surface samples, as described in Section 2.2. Sensor locations are calculated at a specified distance from and approximately normal to this estimated profile, and the antenna is positioned at these locations with the sensor arm [22]. Once the EM measurement is acquired at each location along the profile, the robotic positioning arm is rotated to repeat the process.

To obtain reasonable comfort for patients, scan patterns are designed such that the total scan time is less than 30 min per breast. The laser scan takes approximately 30 s to acquire laser measurements, while each EM measurement takes 5 s to acquire (including the time taken to position the sensor). With 6 EM positions evaluated per profile, the total time to acquire both the laser and EM measurements is 60 s per profile. Patient scans typically involve 20 profiles, as our analysis indicates that this provides sufficient spatial sampling [28,29]. When combined with the current bandwidth of the microwave signal, our measurement strategy provides resolution of approximately 5 mm. Increasing bandwidth to further improve resolution would result in increases in the EM measurement time. However, frequencies greater than 10 GHz typically experience significant attenuation in tissues, imposing a limit on the achievable resolution. With a 20 minute total scan time as a baseline case for comparison, each additional profile adds 1 minute of acquisition time, or increases the scan time by 5%.

As we aim to minimize scan time, this data acquisition strategy involves acquiring surface information very rapidly to take advantage of the high accuracy and resolution of the laser data while minimizing the time between the surface and microwave scans. Acquiring surface measurements dynamically demands that the estimation procedure be robust to the noise levels present in the dynamic data and furnish profiles with an accuracy appropriate for practical antenna positioning. During the measurement process, a limited number of profiles are acquired to reduce the overall time to acquire a full set of EM and laser measurements. Therefore, the surface reconstruction algorithm must provide accurate estimates using this sparse set of surface measurements.

### 2.2. Profile Estimation

**Figure 2:**
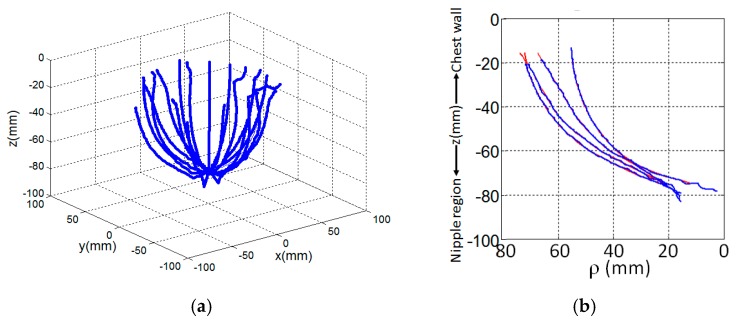
(**a**) Surface samples acquired dynamically over profile by laser as it moves vertically from chest wall to nipple region at a given azimuth. Process repeated over equally spaced azimuths. (**b**) Breast profile estimated (red) from these measured data (blue) at four different azimuth angles of a patient. An extrapolation of the profile is shown near the chest wall.

In the prototype described in Section 2.1, a laser scans vertically along a breast profile *Γ_i_* at a given azimuth angle *ϕ* (Figure 1c). This is repeated for *M* equally spaced angles, *Δϕ*, around the object of interest. The example in Figure 2a shows surface samples measured from 20 profiles, each separated by 18 degrees. Profile samples may also be acquired with EM measurements, such as with the multi-static antenna array described in [11,12,13]. Use of either EM or laser measurements pose a significant challenge when estimating the individual profiles because the data are noisy (from e.g., differential shaking of laser as it scans the breast, rapid vertical movement of the laser to acquire dynamic measurements, sloping surfaces, reflections from the environment).

The breast profile is estimated by fitting a basis of piecewise cubic spline functions to the measurements using a least squares approximation technique [30]. The procedure is selected due its smoothing effect on the approximation since the data are typically contaminated by a significant amount of measurement noise. The degree of smoothing is controlled by selecting an appropriate number of knot variables to delineate the profile into regions. Although the number and placement of knots may be optimized using [31], an excessive number of knot variables may lead to an overly complex model that overfits the data. In this case, the model of the profile can exaggerate minor fluctuations in the data. To avoid this scenario, the goal of the model is to represent the underlying structure of the data without modeling minor fluctuations. It is determined heuristically that this condition is satisfied with six knot variables evenly distributed along the acquired profile; this is explored in more detail in Section 3. The result of the spline fit is a curve ${\hat{\Gamma }}_{i}$ that models the breast surface geometry (see Figure 2b for examples). This process is repeated for *M* azimuth angles. Extrapolation is applied such that all profiles extend to the same maximum location along the *z*-axis. For the *i*th profile, the maximum and minimum heights of the sensor data are calculated as:

(1) $$zMax_{i}=\underset{z}{\max}\left({\left\{z_{j}\right\}}_{j=1}^{N}\right),$$

and:

(2) $$zMin_{i}=\underset{z}{\min}\left({\left\{z_{j}\right\}}_{j=1}^{N}\right),$$

where *z_j_* is the *z*-component of each point of the data associated with the *i*th profile, *j* is a surface sample of the *i*th profile, and *N* is the number of measured surface samples acquired with the sensor over the *i*th profile. The maximum extent over all *M* profiles is:

(3) $$z_{Max_{0}}=\underset{zMax_{i}}{\max}\left({\left\{zMax_{i}\right\}}_{i=1}^{M}\right).$$

The *i*th profile is extrapolated from $zMax_{i}$ to $zMax_{0}$ when $zMax_{i}<zMax_{0}$ using a basis of spline functions that have second order of smoothness at the new breaks (or knot locations) in the region of extrapolation. Examples of extrapolated regions are shown in Figure 2b.

The *i*th estimated profile, ${\hat{\Gamma }}_{i}$*,* is uniformly sampled to form an *R*-by-*1* vector, ${\hat{PF}}_{i}$ Formation of ${\hat{PF}}_{i}$ allows the quality of the estimated profile to be evaluated using metrics described in Section 2.3. Samples are also used to reconstruct an estimate of the surface.

### 2.3. Surface Estimation

The surface model is constructed by forming contours at different heights along the object with points extracted from the estimated profiles. The difference in height between each contour is calculated so that the contours are evenly distributed over the *z*-axis. The extent, *z*_0_, along the *z*-axis used to calculate the separation between contours is the same for each profile:

(4) $$z_{0}=\underset{zMax_{i}}{\max}\left({\left\{zMax_{i}\right\}}_{i=1}^{M}\right)-\underset{zMin_{i}}{\max}\left({\left\{zMin_{i}\right\}}_{i=1}^{M}\right).$$

A total of *K* samples from each profile are acquired over extent *z*_0_ to form *K* contours, so the contours are separated by $∆z=z_{0}/\left(K-1\right)$. The *k*th contour is formed with *M* points, i.e., a point from each of the *M* profiles. The contour points are preprocessed to remove any self-intersecting curves, since it is assumed that the data are extracted from surface points that form a simple and closed contour. The contour is also assumed to be smooth, so subsequent to these pre-processing steps, smooth breast boundaries in the noisy data are recovered using the technique described in [32]. After computing interpolating points between the *M* points, splines are fitted to form a contour model. Examples demonstrating how contours are constructed with this approach are provided in Section 3.

Each contour is then uniformly sampled such that the distance between samples is approximately equivalent to *Δz*. The surface is therefore approximated with the cloud of estimated surface samples, ${\hat{P}}_{S}$; these points are used to evaluate the quality of the surface model using metrics described in the next section. 

**Figure 3:**
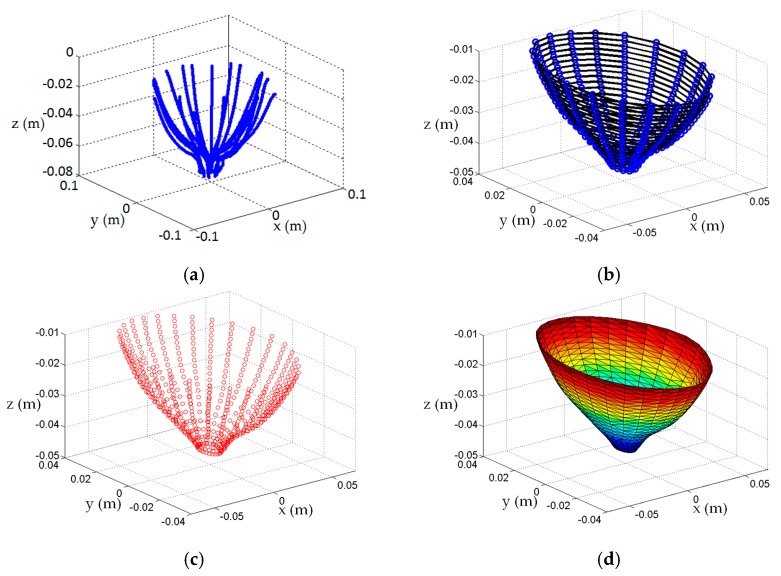
Sequence of steps to construct breast surface of patient: (**a**) Laser measurements, (**b**) 20 profiles estimated from the laser data are sampled (blue dots) at 22 different heights. For each height, a contour (black curve) is formed from the 20 profile samples to form 22 contours; (**c**) Points on contour are preprocessed to remove any self-intersecting curves, interpolating points are calculated between points, and splines fit the data to form a smooth model of contour. Contours are uniformly sampled to form a cloud of points (red dots),$\;{\hat{P}}_{S}$, that estimate the surface; (**d**) Tessellated surface model of breast formed from surface samples.

When an algorithm (e.g., [21]), is applied to these samples to produce a surface mesh and an approximate medial axis, the surface is tessellated into equilateral triangles. Hence, setting the density of the profile sampling with an appropriate value for *K* determines the separation between contours, *Δz*. This, in turn, establishes the size of the equilateral triangles of the tessellated surface. Therefore, the density of tessellation may be adjusted with *K* to suit the application. For radar-imaging (e.g., [3]), a coarsely tessellated surface is required to facilitate the calculation of path lengths in the various materials during image formation. Conversely, a finely tessellated surface is more appealing for microwave tomography (e.g., [14]), or for assessing variables associated with EM data acquisition such as penetration depth and object coverage [16]. An example showing the steps used to construct a surface model of a patient is shown in Figure 3.

### 2.4. Evaluation of Techniques

**Figure 4:**
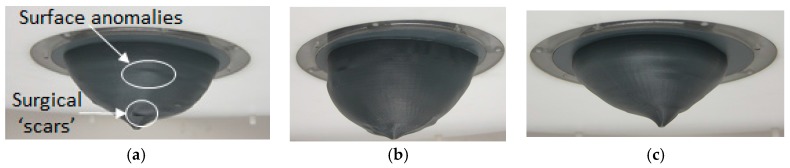
Surface models fitted into top lid of the prototype shown in Figure 1. Model A, B, and C in (**a**–**c**), respectively.

The surface reconstruction methods are first evaluated using anthropomorphic breast phantoms. This is accomplished using measurements from patient scans. Specifically, three models (A, B, and C) are constructed from laser data acquired during the patient study reported in [3]. We note that the laser and scanning approach are different than those described in Section 2.1, as static measurements were acquired from an earlier prototype (cf. [9]) than used for the present study. The reconstruction techniques are applied to each set of laser measurements to form surface models and an orientation flange is added to each model. Plastic anthropomorphic breast phantoms are printed with a 3D printer (Replicator 2, MakerBot Industries, Brooklyn, NY, USA). This material is chosen as the models are conveniently printed with a 3D printer. Past studies using various materials (e.g., metals, wood, etc.) have demonstrated that the material of the object does not significantly impact the accuracy of the results. The experimental models are shown in Figure 4 and demonstrate the variety of sizes and shapes of the three models used in the evaluation. Small variations of the surfaces (e.g., surgical ‘scars’) are also observed in Figure 4a.

As described in Section 3.3, numerical models are also generated to permit testing with EM data. These numerical models have the same shapes as the experimental models, however are assigned dielectric properties of biological tissues as in [16].

The experimental models are scanned with the prototype system. For each vector of points estimated from experimental data, ${\hat{PF}}_{i}$, *R* samples are extracted from the corresponding profile of the numerical model. This forms an *R*-by-*1* vector **PF*_i_***. Comparison of estimated (${\hat{PF}}_{i}$) and reference (**PF*_i_***) profiles permits evaluation of the quality of the estimated profile. Likewise, the estimated surface samples, ${\hat{P}}_{S}$, of an experimental model are compared with the corresponding surface samples, **P_S_**, of the reference model (i.e., surface samples of the numerical model). Similar approaches are followed for points estimated from the numerical EM data. The metrics used to evaluate the quality of the estimated profiles and surfaces are described next.

First, the similarity of the reference profile to the estimated profile is measured with the goodness of fit metric. This is given by:

(5) $$GOF{\left(PF,\;\hat{PF}\right)}_{2}=\;1-{\left\|\frac{PF-\hat{PF}}{PF-\mu_{\mathrm{ref}\_\mathrm{profile}}1}\right\|}_{2},$$

where $GOF{\left(\cdot ,\cdot \right)}_{2}$ indicates that the metric is applied to the profile over ${\mathbb{R}}^{2}$; $\|\cdot {\|}_{2}$ denotes the *L*_2_-norm on ${\mathbb{R}}^{2}$, µ_ref_profile_ is the mean value of the reference profile evaluated over ${\mathbb{R}}^{2}$, and; **1** is a vector of ones having the same length as **PF**. The GOF costs vary from –infinity (bad fit) to 1 (perfect fit).

The similarity between the estimated and reference surface points is also evaluated. The discrepancy between surface points ${\hat{P}}_{S}$ and **P_S_** is measured as:

(6) $$Discrepancy\left(P_{S},\;{\hat{P}}_{S}\right)=\;\frac{\sum_{i=1}^{n}\sum_{j=1}^{p}{\left(P_{S\left(i,j\right)}-{\hat{P}}_{S\left(i,j\right)}\right)}^{2}}{\sum_{i=1}^{n}\sum_{j=1}^{p}{\left(P_{S\left(i,j\right)}-mean\left(P_{S\left(:,j\right)}\right)\right)}^{2}},$$

Consequently, the similarity measure is simply:

(7) $$Similarity\left(P_{S},\;{\hat{P}}_{S}\right)=\;1-\;Discrepancy\left(P_{S},\;{\hat{P}}_{S}\right).$$

The similarity measure gives a value between 0 and 1 to describe the similarity between the reference and estimated surfaces. Values near 0 imply dissimilarity, while values near 1 imply similarity. The distance between each point in the reference set and the corresponding point in the measured set is computed with:

(8) $$d\left(P_{S\left(i,j\right)},{\hat{P}}_{S\left(i,j\right)}\right)=\|P_{S\left(i,j\right)}-{\hat{P}}_{S\left(i,j\right)}\|.$$

The mean error and variability of error over all points on the surface are then evaluated. For a given azimuth angle, the distances measured are organized in descending order along the *z*-axis. Repeating this procedure over the range of azimuth angles leads to the formation of a 2D error map that shows the measured distance between a point on the estimated surface and the corresponding point on the reference surface. The maps furnish insight into how the individual errors are distributed over the breast surface.

## 3. Results

First, we evaluate profile estimation with laser data collected using the 3 different models. Next, surface estimation is explored with these models and the impact of including different numbers of profiles is examined. Surface estimation is then applied to points acquired with EM data. Finally, the techniques are applied to create surface estimates of patients.

### 3.1. Profile Estimation Results

**Figure 5:**
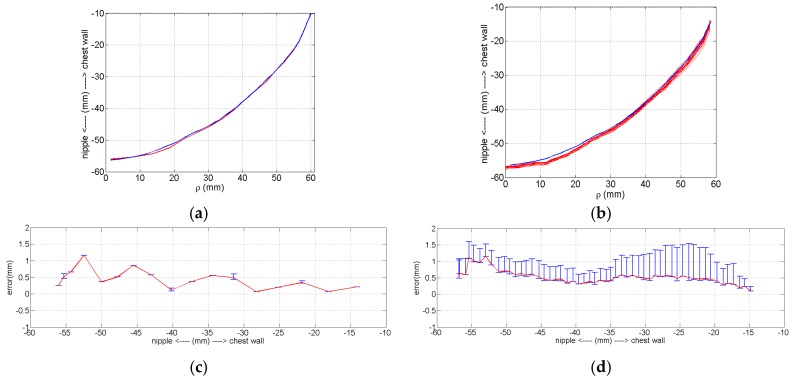
Comparing accuracy and precision between static and dynamic measurements of model B. (**a**) Reference profile (blue) and three set of static measurements (red). (**b**) Reference profile (blue) and 15 sets of dynamic measurements (red). (**c**) Mean static measurement error and max/min error bars. (**d**) Mean dynamic measurement error and max/min error bars.

Two options are available for data acquisition with the laser: collecting measurements at selected locations along the *z*-axis with the positioning arm static, or collecting measurements dynamically while the laser is scanned at 30 mm/s. The dynamic approach facilitates more rapid data collection, however is expected to result in greater error. To quantify this error, we compare three sets of static measurements (Figure 5a) and 15 sets of dynamic measurements (Figure 5b). The errors for the two approaches are shown in Figure 5c,d, demonstrating that the static measurement approach is more precise. Overall, the mean error for the static and dynamic profiles is 0.43 and 0.52 mm, respectively, and indicates comparable, sub-mm accuracy of the static and dynamic measurements. A loss of accuracy is apparent toward the nipple region of the breast, which is expected due to the sloping surface. For dynamic data, the location of the robotic arm is used as a reference in order to accurately transform each surface sample to a point in 3D space. Since the arm is moving, its speed and acceleration are taken into account when calculating its position. Inaccuracies in the assumed speed and acceleration of the laser used for these calculations contribute to the errors in the measurements shown in Figure 5b. However, dynamic data are acquired with the prototype described in this manuscript due to the requirement for patient-specific EM sensor positioning. The use of dynamic data and the presence of the errors is a key motivation for developing the method presented in this manuscript that is robust to noisy data.

**Figure 6:**
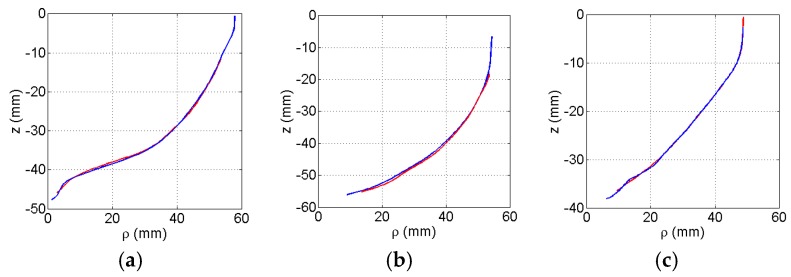
Estimated profile (red) fitted to measured laser data plotted with reference profile (blue) of numerical model for: (**a**) A, (**b**) B, and (**c**) C.

**Figure 7:**
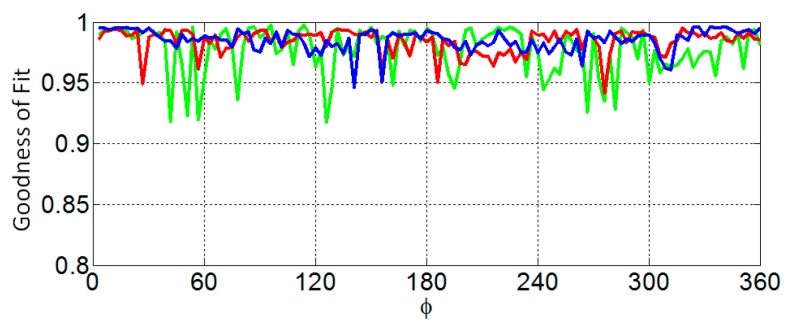
Goodness of fit of profile as the azimuth angle varies for models A (blue), B (red), and C (green).

To assess the performance of the profile estimation with different breast shapes, the breast models are positioned in the prototype system and scanned with the laser. For a given azimuth angle (*ϕ*), a measurement is acquired by scanning the model vertically along the *z*-axis. The azimuth angle is changed and the process is repeated so that 120 equally distributed profiles are collected. Each profile is fitted to the laser data using the methodology described in Section 2.2. The profiles are estimated with 6 knot variables evenly distributed along the acquired profile. An example of the estimated and corresponding reference profile for each model is shown in Figure 6. For these examples, the estimated profiles follow the shape of the reference profiles. The GOF between the estimated and reference profiles are summaries in Table 1. Finally, reference profiles for each of the three models is plotted in Figure 7 over the range of azimuth angles.

Figure 7 suggests that the profiles estimated with the laser measurements closely match the reference profiles, demonstrating high performance for this set of models. This is further supported by mean GOF values close to 1 and low variability values evaluated for each model (Table 1). The mean distance error values are also shown in Table 1. Because the tip of an EM sensor is positioned approximately 5 mm from the skin, we aim for 1 mm error maximum. This ensures that the sensor does not touch the skin as a result of error in profile estimation. Therefore, the sub-mm mean distance error values that are shown indicate that this criterion is met.

The number of knot variables used to model the profiles is progressively increased from six to a maximum of 22. For these measured data, increasing the number of intervals does not impact the mean similarity values. This implies that the underlying structural information contained in the measurements is extracted. However, small spatial variations of the surface are not provided by the dynamic laser measurements. Furthermore, the measurement noise level may obscure detailed surface information.

### 3.2. Surface Estimation Results

**Figure 8:**
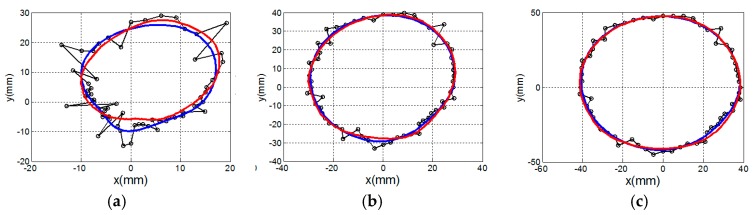
Contour of surface model estimated at three different heights for model C. Estimated contour (blue) fitted to laser points (black dots connected with black lines). Actual contour is represented with red line. Contour near: (**a**) nipple region, (**b**) middle of model, and (**c**) chest wall.

Scans of the three models are used to construct surface estimates using the method described in Section 2.3. First, several contours of model C are shown in Figure 8 to illustrate discrepancies between the actual surface, measured sampled points, and the surface estimate. A loss in accuracy is observed near the nipple region and an improvement is observed near the chest wall.

**Figure 9:**
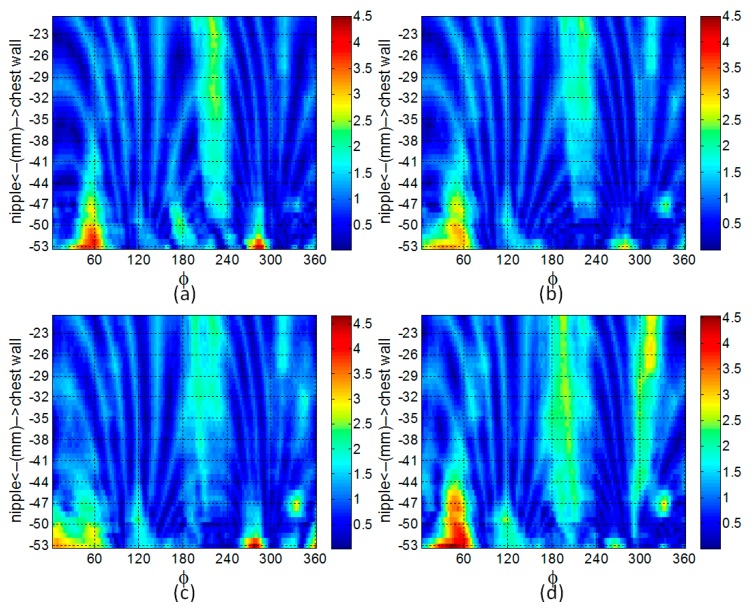
Map showing distance errors (in mm) between the estimated and reference surfaces for model A. The surface is estimated with (**a**) 120, (**b**) 60, (**c**) 20, and (**d**) 15 profiles.

**Figure 10:**
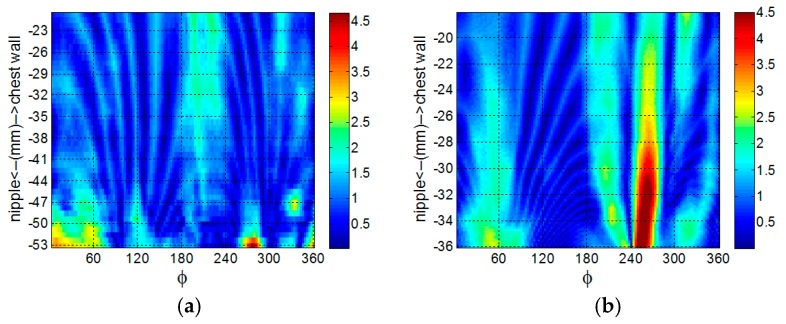
Map of distance error (in mm) over surface estimated with 20 profiles of: (**a**) model B, and (**b**) model C.

Next, we explore the performance of the surface estimation algorithm by examining the impact that the number of profiles has on the quality of the estimate. Surfaces are estimated with 120, 60, 20 or 15 profiles. The similarity and distances between the estimated and reference surfaces is measured with Equations (7) and (8), and is summarized for model A in Table 2. The error map constructed using the methodology described in Section 2.4 is shown in Figure 9. The process is repeated for models B and C and the results are shown in Figure 10 and Table 3 and Table 4, respectively. For each model, decreasing the number of profiles from 120 to 20 does not significantly impact the quality of the surface estimation, as the similarity measure and mean distance error remain comparatively constant. Although reducing the number of profiles further to 15 has no appreciable impact on the quality of surface estimates for models A and B, a drop in similarity and corresponding increase in mean distance error are observed for model C. This is discussed further in Section 4. We note that when scanning a patient, the protocol used at the University of Calgary acquires 20 profiles separated by 18 degrees. This protocol is expected to provide laser data that enables a sufficiently accurate estimate of the breast surface, as well as appropriate illumination with EM signals [28,29].

In order to illustrate the impact of incorporating surface estimates into images, we reconstruct microwave radar images of a simulated breast model (model 2 in [16]). The breast model consists of fat, a tumor, and skin layer. The model and sensor are placed in an immersion liquid of canola oil. The image formation algorithm is described in [16] and includes a step to reduce reflections from common structures such as skin (clutter).

**Figure 11:**
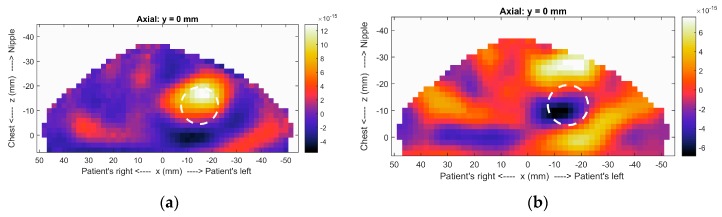
Radar images of realistic breast model formed (**a**) with and (**b**) without surface estimate. In (**b**), the image is limited to the breast region in order to enable comparison. The circle indicates the actual location of the tumor. Both images are formed with 2 mm voxel size, so the outline of the breast is not indicative of the surface estimate.

Because radar images indicate locations where dielectric properties change, responses in the images correspond to the tumor and clutter. Images are formed for two scenarios: one in which the breast surface is estimated and one in which the breast surface is unknown. In the former scenario, specific paths are calculated through the immersion liquid (*ε*_r_ = 2.4), skin (*ε*_r_ = 36) and breast tissues (*ε*_r_ = 4.6). In the latter scenario, constant dielectric properties (*ε*_r_ = 5) are assumed for all three regions. The images shown in Figure 11 indicate that the tumor is accurately detected when the surface estimate is incorporated. When the different materials are not considered, the error in travel time calculations results in lack of coherent summation of the tumor response. For patient scans, prior knowledge of tissue properties is not typically available. In [33], an algorithm that estimates these properties using characteristics of responses in the image is reported. We note that the images in Figure 11 are formed with property estimates of the breast tissues that are very similar, highlighting the need to include the surface estimate to ensure accurate imaging.

In the context of microwave tomography, we have collaborated with the Electromagnetic Imaging Laboratory at the University of Manitoba to investigate the impact of incorporating surface estimates in the reconstruction algorithm. The results of the study are reported in [14] (e.g., Figure 17 page 12). Without the breast surface incorporated into the image domain, internal structures are not clearly resolved. Namely, the glandular structure appears blurred, the fat region cannot be clearly distinguished from the gland region, and the dielectric properties are generally not accurately reconstructed. Incorporating the skin surface into the image domain leads to a significant improvement in the image quality in terms of being able to resolve internal features and structures within the breast, and there is a substantial enhancement in the accuracy of the dielectric properties that are reconstructed.

### 3.3. Comparing Surfaces Estimated with EM and Laser Data

Several microwave imaging prototypes incorporate arrays of microwave sensors that are placed in hemispherical cups that contact the breast [11,12,13]. It is not possible to include a laser scan with these prototypes, however surface estimates are still important for image formation for reasons described in the Section 1. In this section, we demonstrate the application of the profile and surface estimation algorithms to these scenarios. Our research team has constructed breast phantoms as described in [34], however constructing anthropomorphic phantoms with materials appropriate for microwave frequencies is challenging.

The experimental phantoms that we have constructed are symmetrical, and have a simple smooth surface that is not suitable for comparing the two modalities. Hence, to provide a relevant framework that may be used compare the surface estimated from data generated from laser and electromagnetic sensors, we opted for simulations of models with the same geometry but properties representative of tissues (similar to [16]).

The numerical electromagnetic breast models used for this study are the same as those described in [16]. The model of the ultra-wideband antenna used for the numerical study is reported in [26], and operates over a frequency range of a 2.4 to 18 GHz. For this study, the antenna is sequentially scanned around the model to 300 positions. At each position of the scan, the antenna is excited with a differentiated Gaussian pulse having a −3 dB bandwidth of 4.14 GHz (1.45–5.59 GHz), and the corresponding reflection data are collected. Both model and antenna are immersed in a material representing canola oil, and simulated with a 3D FDTD solver (SEMCAD X, SPEAG, Zurich, Switzerland). The corresponding reflection data are collected by sequentially scanning the ultra-wideband antenna to 300 different positions around the model in a hemispherical scan pattern as presented in [16] to emulate a multi-static system (e.g., [12]).

The surface of the outer skin layer is estimated from these reflection data using the procedure described in [35] (p. 2139). The technique is summarized as follows: (1) A calibration is first performed to remove the contributions from the antenna and the external environment, (2) the calibrated data are normalized to the reflected signal’s maximum positive value, (3) the reflection from the surface is identified by modeling the backscattered data received by a sensor as a superposition of attenuated and delayed replicas of a reference signal plus noise, (4) this signal model is incorporated into a decomposition technique described in [36] to identify the reflection from the surface and to estimate the time-of-arrival of this reflection, (5) the time-of-arrival is used to estimate the distance from the surface to the antenna aperture, (6) the point on the skin surface is mapped from the known antenna aperture coordinates using the known orientation of the antenna with respect to the surface and the estimated distance. Repeating (1)–(6) for all *N* antennas (in this case 300 array elements) in the array leads to a set of *N* surface samples.

The profile and surface estimation techniques are then applied to these surface samples to emulate scenarios whereby microwave sensors are integrated in hemispherical cups. Each profile is estimated from 10 electromagnetic (EM) surface samples. Using the same number of knot variables as is used for the laser data (i.e., six), the profile would be delineated into eight intervals. This represents an excessive number of knot variables relative to the number of samples, and may lead to an overly complex model that overfits the data [30]. In this case, the model of the profile can be influenced by noise present in the data. As a remedy, we chose to use just three knot variables distributed over the profile to fit a curve to the 10 surface samples to afford a degree of smoothing of the data.

**Figure 12:**
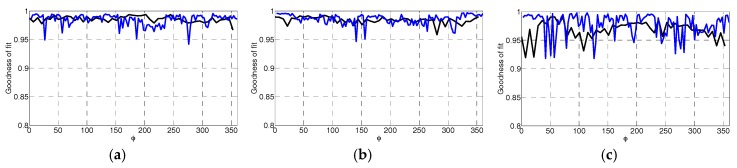
Comparison between the goodness of fit of profile as the azimuth angle varies between profile estimated with electromagnetic (black) and laser (blue) surface samples for models A (**a**), B (**b**), and C (**c**).

**Figure 13:**
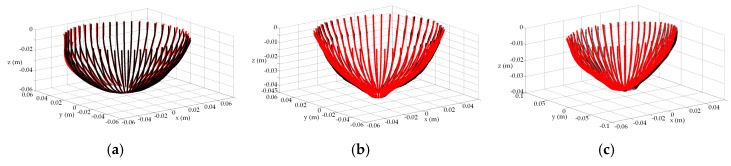
Contours of surface model are uniformly sampled to form a cloud of points (red dots andblack dots) corresponding to the estimated surface from the electromagnetic and laser data, respectively. Surface estimates of models (**a**) A, (**b**) B, and (**c**) C.

The similarity of the reference profile to the estimated profile is measured with the goodness of fit (GOF) metric. A plot of the GOF of the profiles estimated from EM and laser data as the azimuth angle varies is shown in Figure 12. In Table 5, the mean and variance of the GOF over all profiles is compared with the corresponding values over the profiles estimated with laser data. The surfaces are estimated from 50 profiles that are each separated by 7.2°. The point clouds generated from surfaces estimated from EM and laser data are shown Figure 13 as red and black dots, respectively. The similarity metric given by Eequations (6) and (7) is applied to the point cloud generated from the surface estimated from EM data and the point cloud generated from the corresponding reference surface. The similarity of the surfaces estimated from the EM data with the appropriate reference surface are 0.981, 0.983, and 0.941 for models A, B, and C, respectively, demonstrating comparable performance to the results obtained with laser measurements (Table 2, Table 3 and Table 4).

We note that 300 points were used for constructing the surface with EM data. For measurement scenarios described in [12] with a multi-static array incorporated into a ceramic cup, 101 surface points are reported for reconstructing the surface [17]. A degradation of performance of the surface estimation is expected with a reduced number of points. Our goal was not to demonstrate the advantage of acquiring surface samples with lasers compared to EM sensors. In the case of the measurement systems comprised of a ceramic cup, it is not practical to use a laser to acquire the surface samples. Likewise, for the monostatic measurement system developed by the University of Calgary and described in this manuscript, it is not practical to estimate the profile with EM samples, as an estimate of the profile is necessary for patient specific EM sensor positioning. Therefore, the goal of Section 3.3 is to demonstrate the flexibility of the surface estimation algorithm to be applied to data from a variety of modalities.

### 3.4. Application to Patient Data

**Figure 14:**
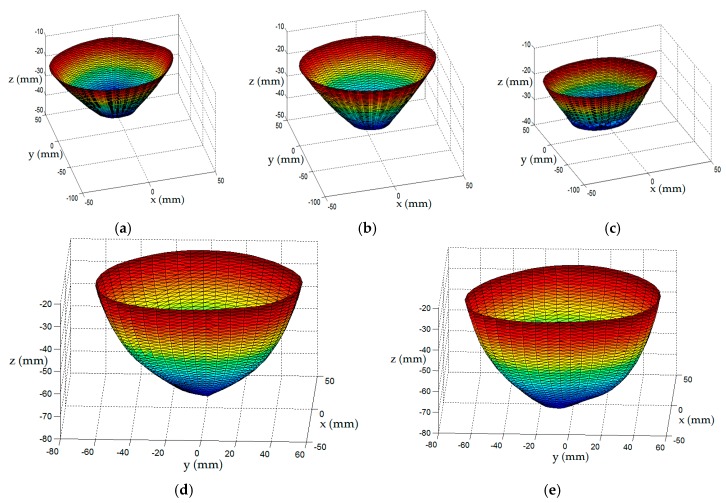
(**a**) Surface of left breast constructed from volunteer A. Surface reconstructed of same breast 4 weeks later, and 6 weeks later, (**b**), and (**c**), respectively. (**d**) Surface of left breast constructed from volunteer B and (**e**) surface of same breast 2 weeks later.

In addition to defining the region of interest for imaging, the surface estimates may also be used in assessing repeatability of scans. We performed a study involving microwave breast scans of volunteers and patients (study E-24098 as approved by the University of Calgary Conjoint Health Research Ethics Board). In one aspect of this study, volunteers are scanned multiple times at different intervals in order to assess the repeatability of the imaging technique. For this scenario, the surface model constructed from the laser data may be used to compare the breast shape and position within the prototype between visits. For example, Figure 14 shows the surface estimates for the left breast of two volunteers scanned on different occasions.

For Volunteer A, the duration between the first and second scan was 4 weeks, and the duration between the second and third scan was 2 weeks. For this example, the centroid of each surface model is evaluated. Using the first scan as a reference, the difference in centroid location between the first scan and the subsequent scans is evaluated. The reconstructed surfaces of the second and third scans are then translated by this difference, and a cloud of points is extracted from the three surfaces and compared. Using the measure described in Equations (6) and (7), the similarity between the first and second scan is 0.977, while the similarity between the first and third scan is 0.909. The apparent similarity between the reconstructed surfaces of the first two scans implies that, for both visits, the breast is located in approximately the same position and orientation within the prototype, and has the same general shape. This is crucial spatial information since similarity in the corresponding scattered fields is also expected. Accordingly, differences in the scattered fields can be more strongly attributed to compositional changes within the breast than to changes in the location or shape of the breast. The decrease in similarity between the surfaces reconstructed from the final scan and the previous two visits suggests that there is a change in breast shape compared to the prior visits. This change may be attributed to a change in the volunteer’s position within the prototype.

The surfaces reconstructions of the two scans for volunteer B are evaluated in the same manner. Namely, there is significant similarity between the surface reconstructions for the two scans (0.954).

**Figure 15:**
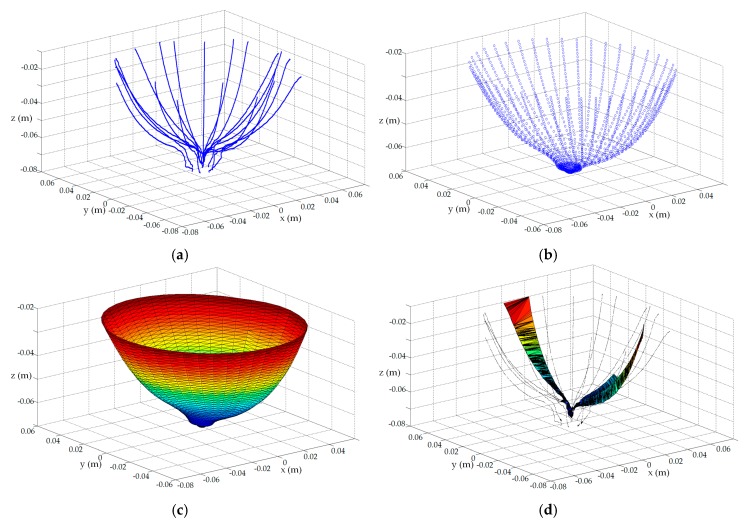
(**a**) Dynamic laser measurements acquired from patient C; (**b**) Point cloud generated with surface estimation algorithm (Section 2.3); (**c**) Surface mesh formed with tessellation algorithm applied to point cloud shown in (**b**); (**d**) Failed surface mesh when tessellation algorithm applied directly to dynamic laser measurements shown in (**a**).

Lastly, the surface reconstruction of a single scan for volunteer C is evaluated to demonstrate the utility of the surface estimation algorithm when it is applied to patient laser measurements. Since patient specific EM sensor positioning is required, the profile estimation algorithm is applied on-the-go to each profile of dynamic laser data to evaluate the EM sensor positions. The full set of dynamic laser measurements are shown in Figure 15a. The surface estimation algorithm (Section 2.3) is applied to these data to generate the point cloud shown in Figure 15b. Comparing the raw measurement and preprocessed point clouds reveals that the surface estimation algorithm smoothens the noisy surface samples (refer to Figure 8 for a more detailed example of surface smoothing), and is able to extract surface information between profiles in order to furnish a dense set of surface points. Notably, these data are absent from the original set of measurements. The tessellation algorithm [21] is applied to the point cloud to produce a surface mesh shown in Figure 15c that may be incorporated into an image reconstruction algorithm to form backscatter energy images [3], or dielectric property maps [14].

In contrast, Figure 15d demonstrates that the tessellation algorithm fails to construct a surface mesh when it is applied directly to the laser measurements. This breakdown of the algorithm suggests that there is insufficient information furnished by the raw noisy laser data to allow the tessellation algorithm to construct a surface mesh. Consequently, surface information that is crucial for reconstruction algorithms is not available in this scenario without the application of the surface estimation algorithm to the laser measurements. This critical impediment arose when we were initially applying the second prototype with patient specific sensor positioning to experimental data. This was a key consideration that ultimately motivated the development of the surface estimation algorithm presented in this manuscript to construct a breast surface estimate from a limited number of profiles comprised of noisy surface samples.

## 4. Discussion and Conclusions

Microwave images are improved by providing information on the location of the tissues in the scanner. For microwave tomography, limiting the reconstruction to the breast reduces the number of unknowns and results in images with increased accuracy [14,37,38,39]. Radar-based approaches benefit from accurate estimates of the path lengths between the sensors and breast [3,11,12,13]. However, providing information on the surface of the scanned tissues is a challenge, requiring either incorporation of an additional sensing technology or estimation of the surface from noisy and sparse data sets. In this paper, we described a prototype system that incorporates multiple sensing approaches to provide accurate estimates of the surface. We introduced algorithms to provide these surface estimates, demonstrating successful application to noisy and sparse data.

To test the algorithms, we used the prototype system to scan experimental breast models with shapes based on patient scans. These results demonstrated that the reconstruction algorithm is capable of accurately estimating the surface using a sparse set of measurements in the range typically acquired for a realistic measurement scenario. The results also exposed the possibility that limits exist on the minimum number of profiles that can be used to construct a surface model. For example, the surface of model C is more complicated and has a larger number of local anomalies (e.g., surgical ‘scars’) compared to the other models. It was shown that 15 profiles does not provide an adequate number of samples to capture the variability of this complicated surface. This motivates future development of a methodology to evaluate the spatial dependence between two samples to ensure that rules of geostatistics are not violated when interpolating data to construct a surface model.

Error maps of the constructed surfaces indicated that the maximum error over the surface using 20 profiles is less than 4.5 mm and occurred near the nipple region. We use the constructed surface models primarily for imaging purposes, as the surface model generated with this approach defines the region of interest for radar imaging. Specifically, the reconstructed surface is used to calculate the path traveled from the antenna to the object surface and from the surface to the focal point. An analysis of the impact that surface errors have on the quality of images created with beamforming is reported in [18] and suggests that narrowband beamforming is not overly sensitive to subcentimeter-scale errors in the estimated location of the surface. This work by others is supported by a brief study we carried out by constructing three test cases for which errors of 2.5, 5.0 and 10.0 mm were introduced to the surface used to form the microwave image (imaging procedures described in [16]). No changes in the image quality (clutter related artifacts in image; localization of the scatter corresponding to a lesion) were observed for the 2.5 mm and 5.0 mm test cases. However, the response in the image corresponding to the lesion was offset by 2 mm for the 10 mm error case. These study results collectively indicate that the surface models provide an accuracy within the tolerance of the algorithms used for radar imaging.

The surface reconstruction algorithms are extended to operate on electromagnetic data. Accordingly, profiles and surfaces are estimated from the electromagnetic data and the results presented in Section 3.3 demonstrated that these reconstructions are comparable to those obtained with laser data. This suggests that the estimation approaches provide a general framework that may be effectively applied to data generated from a variety of modalities. The results also suggest that the algorithm is capable of providing accurate results when applied to a very limited number of measurements.

The surface estimation algorithm was applied to laser data acquired from scans of human subjects. The example demonstrated that the algorithm is robust to measurement noise levels typically encountered in patient studies. It is capable of reconstructing surfaces with sufficient accuracy to permit the evaluation of similarity in breast positioning and orientation for repeat visits of a patient. Therefore, the surface estimate is a useful tool for evaluating the repeatability of imaging.

## Figures and Tables

**Table 1:** Mean and standard deviation of profile over 120 profiles.

Model	A	B	C
GOF Mean	0.986	0.984	0.977
GOF Std.	0.009	0.010	0.020
Distance error mean (mm)	0.669	0.666	0.935
Distance error std.	0.535	0.594	0.748

**Table 2:** Error evaluation for surface estimates of Model A.

Number of Profiles	120	60	20	15
Distance error mean (mm)	1.02	1.00	1.18	1.19
Distance error std.	0.51	0.52	0.61	0.66
Similarity	0.980	0.980	0.979	0.978

**Table 3:** Error evaluation for surface estimates of Model B.

Number of Profiles	120	60	20	15
Distance error mean (mm)	0.93	0.90	0.93	0.90
Distance error std.	0.56	0.53	0.56	0.53
Similarity	0.978	0.979	0.978	0.979

**Table 4:** Error evaluation for surface estimates of Model C.

Number of Profiles	120	60	20	15
Distance error mean (mm)	1.00	0.85	0.90	1.68
Distance error std.	0.64	0.50	0.58	1.13
Similarity	0.981	0.983	0.980	0.960

**Table 5:** Mean and standard deviation of profile GOF over 120 profiles.

Model	EM Data		Laser Data	
	Mean	Std.	Mean	Std.
A	0.985	0.005	0.986	0.009
B	0.984	0.006	0.984	0.010
C	0.968	0.016	0.977	0.020
